# Depression and health-related quality of life of patients with type 2 diabetes attending tertiary level hospitals in Dhaka, Bangladesh

**DOI:** 10.1186/s41256-023-00328-9

**Published:** 2023-10-16

**Authors:** Manish K. Namdeo, Sarita Verma, Rajat Das Gupta, Rubana Islam, Shaila Nazneen, Lal B. Rawal

**Affiliations:** 1 Independent Scholar and Alumni, JPGSPH, BRAC University, Bangladesh, Chhindwara, India; 2 Tata Institute of Social Sciences, Mumbai, India; 3 University of South Carolina, Columbia, SC USA; 4 International Initiative for Impact Evaluation (3Ie), Columbia, SC USA; 5 University of Texas at El Paso, El Paso, TX USA; 6 Central Queensland University, Sydney Campus, Sydney, Australia

**Keywords:** Depression, HRQOL, Comorbidity, Bangladesh, Type 2 diabetes

## Abstract

**Introduction:**

Type 2 diabetes mellitus (T2DM) and depression are closely linked. People with T2DM are at increased risk of developing depression and vice versa. T2DM and depression comorbid conditions adversely affect Health-Related Quality of Life (HRQOL) and management of T2DM. In this study, we assessed depression and HRQOL among patients with T2DM in Dhaka, Bangladesh.

**Methods:**

A cross-sectional study was conducted in two tertiary-level hospitals in Dhaka, Bangladesh. Data were collected from 318 patients with T2DM. A set of standard tools, PHQ-9 (for assessing depression) and EuroQol-5D-5L (for assessing the HRQOL), were used. Statistical analyses, including Chi-square and Fisher's exact tests, Wilcoxon (Mann–Whitney), and Spearman's correlation coefficient tests, were performed using SPSS (v.20).

**Results:**

The majority of the patients (58%) were females, with a mean age (standard deviation) of 52 ± 10 years, and 74% of patients lived in urban areas. The prevalence of depression was 62% (PHQ-9 score ≥ 5). Over three-quarters (76%) reported problems in the anxiety/ depression dimension of EQ-5D, followed by pain/discomfort (74%), mobility (40%), self-care (36%), and usual activities (33%). The depression and T2DM comorbid condition were associated with all the five dimensions of EQ-5D (χ2 statistics with df = 1 was 52.33, 51.13, 52.67, 21.61, 7.92 for mobility, self-care, usual activities, pain/discomfort, and anxiety/ depression dimensions respectively, *p*- < 0.01). The mean EQ-5D index (0.53 vs. 0.75) and the mean EQ-5D VAS (65 vs. 76) both showed lower values in T2DM patients with depression compared to T2DM patients without depression (Wilcoxon test, *p*- < 0.001).

**Conclusions:**

We conclude that the majority of the patients with T2DM had comorbid conditions, and the HRQOL was negatively affected by comorbid depression in T2DM patients. This suggests the importance of timely screening, diagnosis, treatment, and follow-up of comorbid depression in T2DM patients to improve overall health and QOL.

## Background

Type 2 diabetes mellitus (T2DM) is a major public health problem worldwide. Nearly half a billion people are living with T2DM worldwide, of whom 75% live in low- and middle-income countries (LMICs) [1]. The situation is particularly alarming in South-East Asia, where 87.6 million people were affected with diabetes in 2019, resulting in 1.2 million attributable deaths to T2DM [2]. More specifically, Bangladesh follows along with these estimations [3, 4] amidst the aggravating background of a health system where communicable diseases are still prevalent [5]. Bangladesh is among the top 10 countries with the most cases of undiagnosed T2DM in adults [2]. The burden of T2DM involves high costs to patients (US$88 a year) and providers (US$54-64 a year) in Bangladesh [2, 6]. These costs include medical advice, investigations, treatment, travel, productivity loss, and carers' expenses. As reported by the IDF, the annual health expenditure per person with T2DM in Bangladesh during 2019 was estimated at US$64. This figure represents the expenses directly linked to diabetes, irrespective of who pays, whether patients, private or public payers, or the government [2].

Evidence shows that people with T2DM are 2–3 times more likely to develop depression than those without diabetes, and vice versa [7, 8]. In Bangladesh, depression is a major issue, affecting 41% of the general population and 34% of individuals with chronic medical conditions. A study among university students in Bangladesh reported that 65% of patients exhibited depressive symptoms [9]. The co-occurrence of multiple chronic illnesses, such as diabetes and depression, demonstrates a strong interconnection [10], with the odds of depression doubling in T2DM patients [11–13]. Two hypotheses explain this relationship. The first hypothesis suggests that depression precedes diabetes, as changes in counter-regulatory hormones, glucose transport mechanisms, and immune-inflammatory responses contribute to the development of depression [12]. These alterations lead to disruptions in glucose metabolism, resulting in B-cell dysfunction and insulin resistance [12]. The second hypothesis proposes that the chronic nature of diseases like diabetes, along with associated complications, induces stress that can trigger depressive illness in diabetic patients [14]. The relationship between depression and diabetes is bidirectional, and this supports both hypotheses; hence, the validity of one hypothesis does not negate the possibility of the other hypothesis being true [13, 15].

Complications and multi-organ involvement in T2DM, along with high treatment costs, impact the Quality of Life (QoL) of patients and those around them [16]. QoL is an individual's perception of their life within the context of their culture and value systems, considering their goals, expectations, standards, and concerns [17]. QoL encompasses a broader concept of how diseases hinder one's ability to fulfill normal roles, reflected as self-perceived Health-Related Quality of Life (HRQOL) in terms of physical and mental well-being [16, 18, 19].

T2DM itself reduces mean HRQOL scores compared to healthy individuals [20, 21]. The coexistence of depression and diabetes worsens HRQOL, leading to medication non-compliance, increased complications, and elevated suicide risk [21–25]. Assessing HRQOL is valuable in understanding the psychosocial impact of T2DM and coexisting conditions [26–28]. Understanding the psychological dimensions of diseases can lead to improved treatment approaches [29]. Research findings indicate that the incorporation of mental health services into chronic disease care has shown positive outcomes, leading to improvements in both physical health conditions and depression [30, 31].

Studies in Bangladesh on HRQOL have been conducted in various groups, such as adolescents with cerebral palsy [32], leprosy patients [33], older people [34], postpartum mothers [35], T2DM patients [36, 37], and migrant workers [38], but research on T2DM patients with comorbid depression remains unassessed. This study offers unique insights, as it is the first documented study using both PHQ-9 and EQ-5D to assess depression comorbidity in T2DM patients. Considering the ongoing COVID-19 pandemic, mental health support for individuals with diabetes has become even more critical [39]. This further emphasizes the importance of publishing such studies. In this study, we determined the general characteristics and health status (body mass index (BMI), blood pressure, comorbid depression, and HRQOL) among patients with T2DM. We also examined the association between depression and HRQOL.

## Methods

### Study design and setting

This cross-sectional study was conducted between November 2014 and January 2015. A total of 318 patients with T2DM were enrolled in the study at two medical centers in Dhaka, Bangladesh. The first one, BIHS, is a medical teaching institute under the Health Care Development Project (HCDP) of Bangladesh Diabetic Samity, attached to a 250-bed diabetic tertiary care hospital [40], but also linked to smaller regional hospitals and ten urban healthcare centers in and around Dhaka [40]. The second one, DMCH, is a medical college attached to a 2,300-bed hospital [41]. Its Department of Medicine provides outpatient services to diabetic patients on Mondays and Saturdays and is attended by patients from all over the country [41].

### Participants

The study population comprised patients with clinically diagnosed T2DM. The inclusion criteria for the study participants were confirmed cases of T2DM, aged between 30 to 70 years, attending the outpatient departments (OPDs) of the BIHS hospital and DMCH during the study period, and being willing to participate in the study. Pregnant women were excluded, as pregnancy itself may affect the HRQOL in a woman [42]. Similarly, severely ill patients who required emergency care and could not respond to survey questions were excluded.

### Sampling and sample size

Patients attending the OPDs of BIHS and DMCH during the study period were our potential respondents and were conveniently sampled. Patients waiting for their turn for consultation or collecting the results of the routine investigation in the waiting area were requested to participate in the study. 318 T2DM patients (280 from BIHS and 38 from DMCH) were interviewed from the study sites.

The minimum required sample size was calculated using the formula n=Z^2^ pq/d^2^ (Where z = 1.96, p = the proportion of T2DM patients in Bangladesh who reported problems in anxiety/depression dimension of EuroQol-5D (EQ-5D), i.e., 73.6%, q = 1−p, and d = the allowable error of known prevalence, i.e., 5%) [36]. The present study estimated the sample size based on the prevalence of anxiety/depression in EQ-5D-5L dimensions because this was the most relevant dimension associated with the study objective [43]. With a 95% confidence interval and a 5% error, the calculated sample size was 299 [44]. Additional participants were included in the sample size calculation to account for any missing data, resulting in 318 patients being interviewed for the study.

### Study tools

We administered a survey questionnaire written in the Bangla language, comprising four sections: (I) socio-demographic characteristics; (II) physical measurements; (III) EQ-5D-5L for health-related quality of life; and (IV) PHQ-9 (Patient Health Questionnaire-9) for assessing depression (Table 1).

**Table 1:** Study variables and tools

Sections	Variable	Type of Variable	Measurement tools	Assessed information
Section I	Socio-demographic characteristics	Independent	Questionnaire	Age, sex, religion, marital status, education, occupation, income, expenditure, household members, area of residence, age at diagnosis of the illness, and years since the diagnosis
Section II	Physical measurements	Independent	Secondary data from patients’ record	Height, weight, Blood pressure
Section III	Comorbid depression	Independent	PHQ 9	A. PHQ 9 score B. Severity of depression: 1. No; 2. Mild; 3. Moderate; 4. Moderately severe; 5. Severe C. Presence of depression 1. Diabetes with no depression 2. Diabetes with depression
Section IV	HRQOL	Dependent	EQ-5D-5L	A). EQ-5D-5L descriptive system dichotomized for all the five dimensions into the problem and no problem B). Visual analogue scale (VAS) C). EQ-5D-5L index value

To measure the health-related quality of life (HRQOL), we used the standardized EuroQol-5D-5L (EQ-5D-5L) questionnaire. When evaluating HRQOL, different instrument types can be used, including disease-specific, generic, and utility instruments. The EQ-5D is considered the most appropriate generic instrument because of its broad applicability across the population, facilitating HRQOL comparisons [45]. Using EQ-5D provides a simpler, more cost-effective, and comprehensive evaluation of HRQOL, making it valuable for assessing health outcomes in diverse patient populations and conditions. The EQ-5D captures aspects of health, such as mobility, self-care, usual activities, pain/discomfort, and anxiety/depression. It comprises three measurements: (1) the EQ-5D descriptive system, which shows the distribution of individuals across five levels in each dimension; (2) the Visual Analogue Scale (VAS), a continuous value representing general health status; and (3) the EQ-5D index, which assigns a value to each EQ-5D health state, yielding a single composite index [46, 47]. To assess depression levels, we utilized a previously validated Bengali version of the PHQ-9, available for free download on the PHQ website [48, 49]. The PHQ-9 consists of nine items on a 4-point Likert scale, with standard cut-off scores used to classify symptoms of depression as minimal/none (0–4), mild (5–9), and moderate to severe (≥ 10) [50].

### Data collection and processing

We pre-tested the survey questionnaire among a similar population at BIHS Hospital prior to data collection. Data was collected through face-to-face interviews conducted by key researchers and three trained assistants. Each interviewer was assigned a unique code (e.g., K011, S012), and the questionnaires were numbered serially. Patients were identified by combining the interviewers' code and questionnaire serial number (e.g., K011-001, S012-199). Each interview lasted 25–30 minutes, with approximately 7–8 sets of questionnaires completed by each interviewer daily, totaling 35–40 interviews. Questionnaires were checked daily for accuracy and completeness, and feedback was provided to interviewers regularly.

Cronbach’s alpha for the PHQ-9 scale was 0.824, and the correlations between the nine items of the PHQ-9 and total PHQ-9 scores were significant at the 0.01 level, ranging from 0.610 to 0.769. Likewise, Cronbach’s alpha for the EQ-5D scale was 0.816. The correlations between five items of the EQ-5D and total EQ-5D scores were significant at the 0.01 level, ranging from − 0.602 to − 0.794.

### Data analysis

**Fig. 1:**
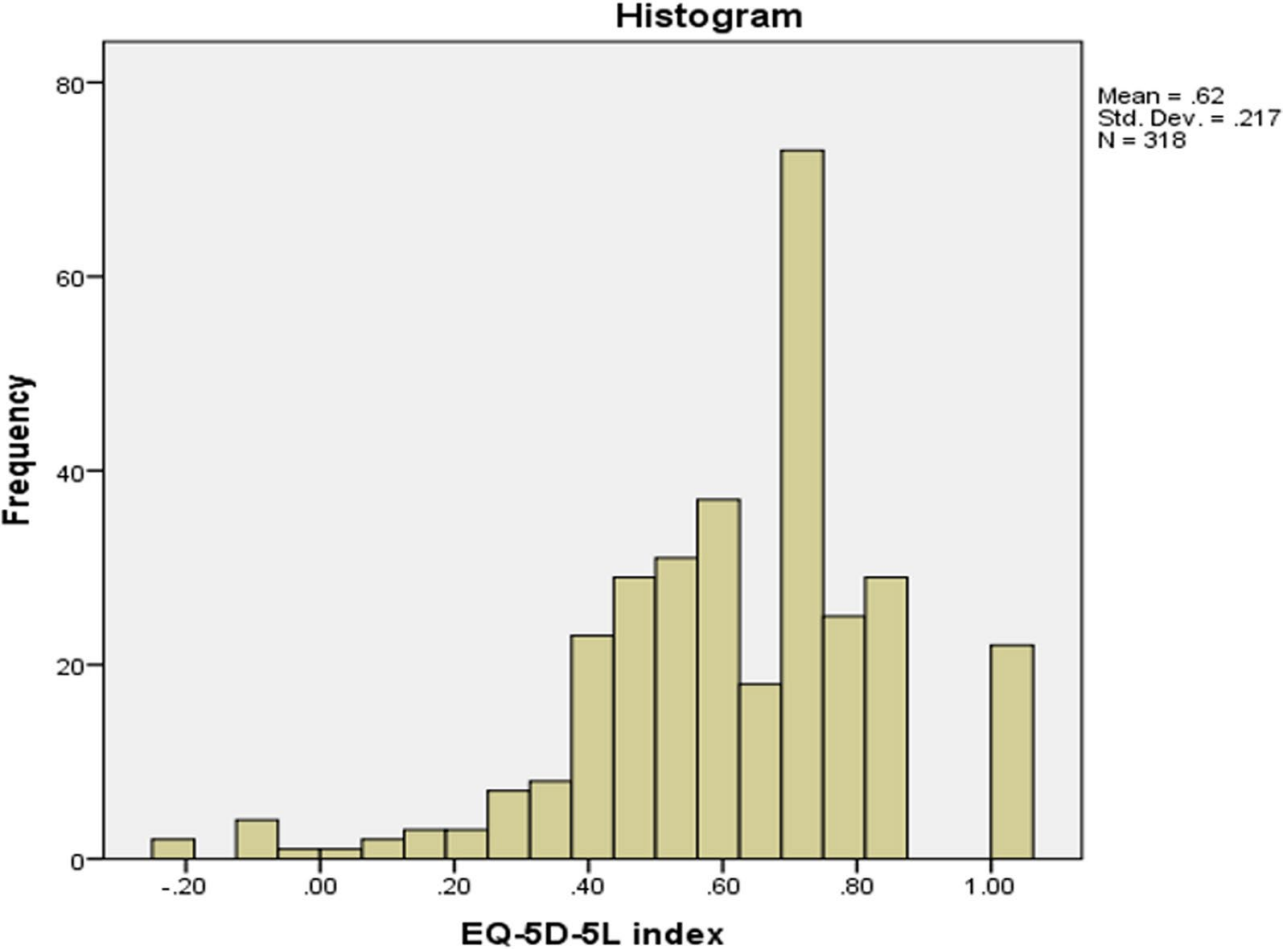
Histogram EQ-5D index: The figure represents a histogram displaying the distribution of EQ-5D index scores in a population. The EQ-5D index is a measure of health-related quality of life, ranging from 0 (representing death) to 1 (representing perfect health). The x-axis of the histogram shows the range of EQ-5D index scores, with the minimum score of 0 on the left and the maximum score of 1 on the right. The y-axis shows the frequency of individuals with each EQ-5D index score

**Fig. 2:**
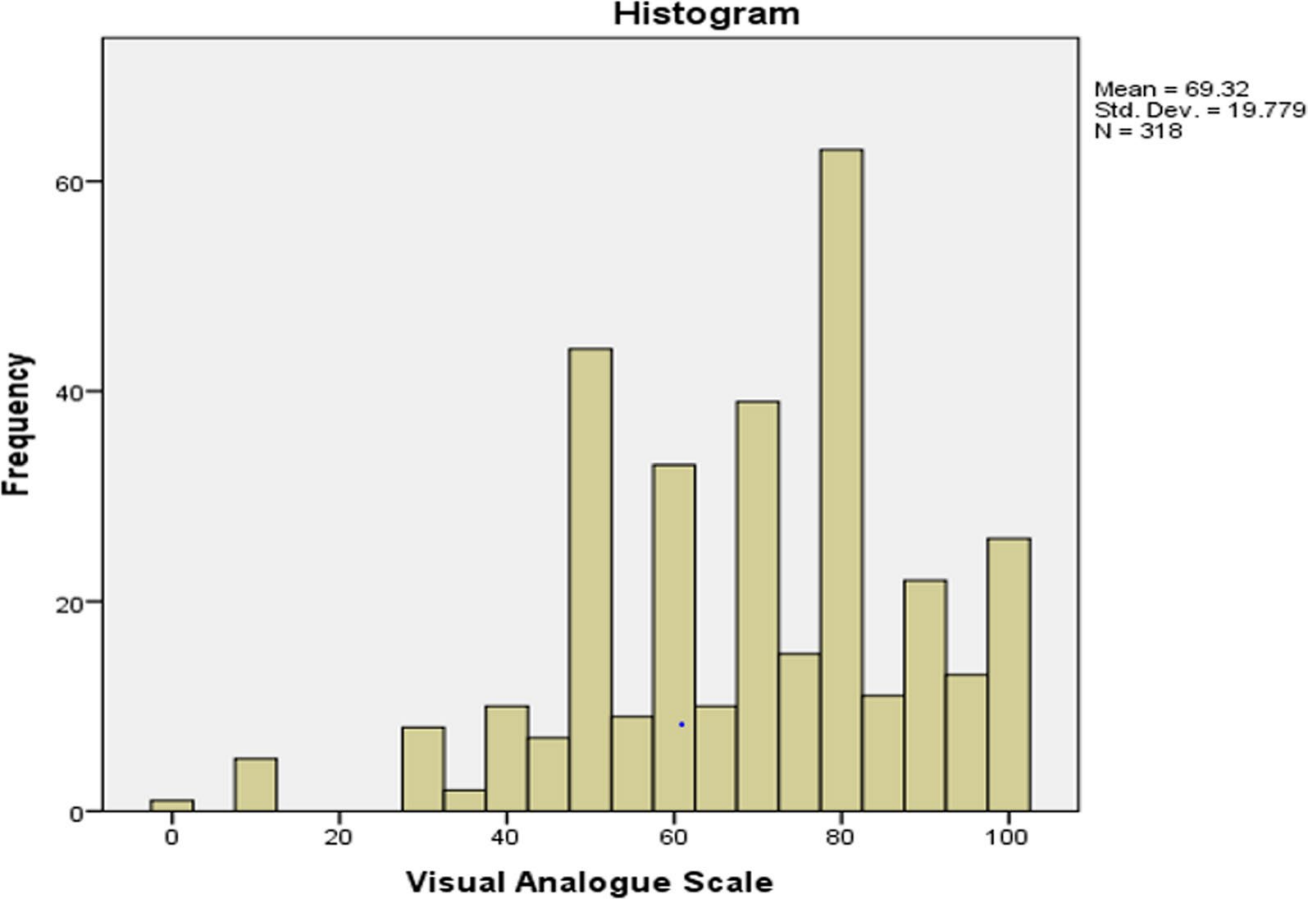
Histogram EQ-5D VAS: The histogram in Figure represents the distribution of EQ-5D Visual Analog Scale (VAS) scores among the study population. The EQ-5D VAS is a measure of health-related quality of life, ranging from 0 to 100, where a score of 100 represents perfect health and 0 represents worst imaginable health state. The x-axis shows the range of EQ-5D VAS scores, and the y-axis represents the frequency of individuals with each score. The histogram is divided into several bars, each representing a range of EQ-5D VAS scores

The data were analyzed using SPSS (v.20) [51]. Outliers in continuous variables were identified using Tukey's fence test, and extreme outliers were described using median and interquartile ranges. Categorical variables were analyzed using frequency tables, Chi-square, and Fisher's exact tests. The EQ-5D index and EQ-VAS (dependent variables) were found to have non-normal distributions with negative skewness in the Shapiro-Wilk test (p < 0.05). The histogram of the EQ-5D VAS revealed multiple spikes corresponding to numbers ending with 0 or 5, indicating a preference for these digits among participants (Figs. 1 and 2). Similar non-normal distributions have been observed in previous studies, warranting the use of non-parametric tests for comparing means [52].

The PHQ-9 questionnaire comprises nine items rated on a 4-point Likert scale. Standard cut-off scores were used to categorize depression as minimal/none (0–4), mild (5–9), and moderate to severe (≥ 10) [50]. In this study, the scores were further collapsed into two categories: no depression (< 5) and depression (≥ 5) [8]. This categorization created two groups among the patients: T2DM patients with depression and T2DM patients without depression. The EQ-5D dimensions were categorized as no problems (level 1) and problems (levels 2 to 5) [53]. Although dichotomizing the EQ-5D dimensions may result in oversimplification of the assessment, it can be useful in standardizing assessments across diverse patients and settings. Additionally, it offers a standardized approach for analysis and enables statistical comparisons. Chi-square, Fisher's exact test, and Wilcoxon (Mann-Whitney) tests were used to examine associations and differences between variables. Significance was set at p < 0.05. Spearman's correlation coefficient assessed relationships between patient characteristics, PHQ-9 scores, and the EQ-5D index. A trend plot was developed to illustrate the relationship between depression levels and the HRQOL index.

## Results

### Socio-demographic characteristics of the patients

Among the 318 enrolled patients, the majority (n = 184, 58%) were female, and most (n = 280, 88%) were from the BIHS hospital. About three-quarters (n = 234, 74%) of the patients were from an urban area. Most of the patients (n = 303, 95%) were married and Muslims (n = 312, 98%) by religion. Most of the patients were educated, and 12% (n = 38) had no formal education. More than half (n = 176, 55%) of the patients were homemakers. Their median monthly household income was 321.50 USD (interquartile range (IQR) = 450.10 USD). After excluding one extreme outlier with an income of 15432.06 USD per month, the average monthly household income of the patients (n = 317) was 492.36 USD (standard deviation (SD) = 565.89 USD). The average monthly household expenditure of the patients was 407.19 USD (SD = 392.95 USD). The reported pattern showed that the majority of the patients had income (46%, n = 146) and expenditure (49%, n = 155) in the 10,000-30,000 BDT (128.60–385.80 USD) category. The mean age of the patients was 52 (SD = 11) years (median = 52 years). The majority (n = 179, 56%) were in the 41–60 age group. The median age at diagnosis of diabetes was 44 years (IQR = 13) (mean age at diagnosis 44; SD = 10 years). More than half (n = 179; 56.29%) of the patients had a family history of T2DM.

### Anthropometric and biomedical measurements

The mean body mass index (BMI) of the patients (n = 259) was 20.18 (SD = 3.03) kg/meter^2^. The mean systolic blood pressure of the patients (n = 284) was 124.95 (SD = 15.37) mmHg (median = 120; IQR = 10 mmHg), and mean diastolic blood pressure was 78.86 (SD = 7.99) mmHg (median = 80; IQR = 05 mmHg).

### PHQ-9 for assessing depression

The proportion of depression among T2DM patients was 62% (PHQ-9 score ≥ 5). Among all the patients (N = 318), 38% (n = 122) had none/minimal depression (PHQ-9 score 0–4); 30% (n = 94) had mild depression (score 5–9); 23% (n = 73) had moderate depression (PHQ-9 score 10–14); 7% (n = 22) had moderately severe depression (PHQ-9 score 15–19); 2% (n = 7) had severe depression (PHQ-9 score 20–27) (Table 2).

**Table 2:** Percentage of patients in different depression severity groups (N = 318)

Severity of depression (PHQ 9 score)	Count	N (%)
None (0–4)	122	38
Mild depression (5–9)	94	30
Moderate depression (10–14)	73	23
Moderately severe depression (15–19)	22	7
Severe depression (20–27)	7	2

### EQ-5D for assessing health-related quality of life

About 40% of patients had problems in mobility, 36% had problems in self-care, 33% had problems in usual activities, 74% had problems in pain/discomfort, and 76% had problems in the anxiety/depression dimension of the EQ-5D (Table 3).

**Table 3:** Frequency of reported problem by dimensions of HRQOL (N = 318)

Dimensions		Count	N%
Mobility	No problem	191	60
	Problem	127	40
Self-care	No problem	204	64
	Problem	114	36
Usual Activities	No problem	212	67
	Problem	106	33
Pain/Discomfort	No problem	84	26
	Problem	234	74
Anxiety/Depression	No problem	77	24
	Problem	241	76

### EQ-5D index and EQ-5D VAS

The mean EQ-5D index was 0.62 (SD = 0.22) (median = .66, IQR = 0.24). Mean EQ-5D VAS was at 69 (SD = 20) (median = 70, IQR = 25). Within their respective categories, individuals in the younger age group, males, Hindus, those who have never been married, without a family history, belonging to smaller households (less than four family members), having a higher education level, working as laborers, with higher household income, shorter illness duration, without depression, residing in urban areas, and having normal systolic/diastolic blood pressure, exhibited higher EQ-5D index and VAS scores (Table 4).

**Table 4:** Mean EQ-5D index and mean EQ-5D VAS (N = 318)

		N (%)	EQ-5D-5L index Mean (SD)	EQ-5D-5L VAS Mean (SD)
Sex	Male	134(42)	.68(.19)	73(20)
	Female	184(58)	.57(.23)	67(19)
Age groups (years)	30–39	38(12)	.69(.21)	64(18)
	40–49	79(25)	.64(.19)	70(21)
	50–59	100(31)	.62(.19)	72(18)
	60 +	101(32)	.56(.25)	68(21)
Marital status	Never married	7(2)	.67(.25)	71(28)
	Married	303(95)	.61(.22)	69(20)
	Widow/Widower	7(2)	.69(.12)	79(11)
	Separated/Divorced	1(1)	.72	70
Family History of T2DM	No	139(44)	.64(.18)	68(19)
	Yes	179(56)	.60(.24)	70(20)
Household members	< 4 or = 4	145(46)	.64(.21)	70(20)
	> 4	173(54)	.60(.22)	69(20)
Education	No formal education	38(12)	.54(.26)	61(21)
	Primary education	103(32)	.57(.24)	66(21)
	Higher sec. education	108(34)	.64(.18)	72(18)
	College and above	69(22)	.70(.18)	75(20)
Occupation	Unemployed	3(1)	.56(.06)	65(22)
	Service	54(17)	.68(.20)	74(23)
	Business	32(10)	.66(.19)	72(23)
	Laborer	7(2)	.75(.22)	74(11)
	Farming	5(2)	.63(.15)	60(10)
	House maker	176(55)	.56(.22)	67(19)
	Retired	41(13)	.70(.17)	74(16)
Monthly household income (BDT)	< 10,000	52(16)	.53(.26)	63(20)
	10,000–30000	146(46)	.63(.21)	67(20)
	30,000–60000	83(26)	.62(.20)	73(18)
	60,000–90000	11(3)	.69(.17)	81(14)
	> 90,000	26(8)	.64(.19)	79(16)
Monthly household expenditure (BDT)	< 10,000	58(18)	.56(.25)	64(19)
	10,000–30000	155(49)	.63(.21)	68(20)
	30,000–60000	80(25)	.62(.21)	73(19)
	60,000–90000	9(3)	.72(.15)	83(16)
	> 90,000	16(5)	.62(.21)	80(18)
BMI	< 18.5 (Underweight)	9(3)	.62(.20)	66(20)
	18.5 to < 23 (Normal wt.)	83(26)	.62(.20)	72(20)
	23 to < 27.5 (Over wt.)	144(45)	.69(.20)	74(18)
	≥ 27.5 (Obese)	82(26)	.55(.27)	69(27)
Duration of diagnosis	Duration < = 3 years	108(34)	.66(.19)	70(18)
	Duration 3–6 years	53(17)	.62(.20)	69(21)
	Duration 6–9 years	47(14)	.59(.18)	69(18)
	Duration > 9 years	110(35)	.58(.25)	69(22)
Depressive symptoms	No depression (< 5 score)	122(38)	.75(.14)	76(16)
	Depression (≥ 5 score)	196(62)	.53(.22)	65(21)
Area of residence	Urban	234(74)	.63(.21)	71(20)
	Rural	39(12)	.49(.27)	60(21)
	Peri-Urban	45(14)	.67(.15)	70(18)
Systolic BP	Normal SBP	47(17)	.72(.16)	68(23)
	Prehypertension	189(67)	.63(.20)	71(18)
	Stage 1 Hypertension	29(10)	.49(.25)	67(25)
	Stage 2 Hypertension	19(7)	.57(.22)	66(19)
Diastolic BP	< 80 (Normal)	70(24.6)	.67(.18)	73(20)
	80–99 (Prehypertension)	168(59.2)	.62(.21)	69(20)
	90–99 (Stage 1 HTN)	35(12.3)	.57(.22)	73(18)
	> 100 (Stage 2 HTN)	11(3.9)	.63(.19)	50(18)

### Association of socio-demographic characteristics and comorbid depression with HRQOL

The proportion of patients having problems in all five dimensions of the EQ-5D (56 patients having problems in mobility vs. 15 patients with no problem, 51 with problems vs. 11 with no problem in self-care, 48 with problems vs. 9 with no problem in usual activity, 83 with problems vs. 59 no problem in pain discomfort, and 81 with problems vs. 61 no problem in anxiety/depression dimension) was significantly higher among those who showed the presence of depression (PHQ-9 score ≥5) (Table 5). The relationship between the EQ-5D dimensions and various demographic factors is presented in Table 5. The study revealed significant Spearman's correlations (p < 0.01) between the EQ-5D index and various demographic and health-related factors, including age (r = − .21), sex (r = − .27), duration of diagnosis (r = − .12), education (r = .26), and PHQ-9 severity score (r = − .61). Additionally, significant correlations (p < 0.01) were observed between the EQ-5D VAS and several factors, such as sex (r = − .15), monthly income (r = .23), expenditure (r = .22), BMI (r = .15), education (r = .20), and PHQ-9 severity score (r = − .38), as presented in Table 6.

**Table 5:** EQ-5D dimensions by selected demographic characteristics (N = 318)

		N (%)	Mobility	Self-care	Usual activities	Pain/Discomfort	Anxiety/Depression
			Problem%	Problem%	Problem%	Problem%	Problem%
Sex	Male	134(42)	32	26	22	63	73
	Female	184(58)	46	43	42	82	78
χ^2^ (df)			5.946(1)	9.533(1)	14.245(1)	14.151(1)	.887(1)
p-value			.015^*^	.002^*^	.000^*^	.000^*^	.346
Age (years)	30–39	38(12)	26	24	24	58	68
	40–49	79(25)	29	20	23	78	82
	50–59	100(31)	42	36	34	78	77
	60 +	101(32)	51	52	45	71	72
χ^2^ (df)			12.590(3)	22.942(3)	11.291(3)	7.064(3)	3.696(3)
p-value			.006^*^	.000^*^	.010^*^	.070	.296
Householder members	≤ 4	145(46)	32	34	32	72	74
	> 4	173(54)	46	38	34	75	77
χ^2^ (df)			6.289	.490	.101	.188	.577
p-value			.012^*^	.484	.750	.665	.448
Education	No education	38(12)	55	55	53	76	71
	Primary edu	103(32)	50	38	37	83	81
	Higher sec	108(34)	31	34	30	72	75
	College and above	69(22)	29	25	23	59	72
χ^2^ (df)			15.168(3)	10.300(3)	10.818(3)	12.576(3)	2.207(3)
p-value			.002^*^	.016^*^	.013^*^	.006^*^	.531
Depressive symptoms	No Depression	122(38)	15	11	9	59	67
	Depression	196(62)	56	51	48	83	81
χ^2^ (df)			52.332(1)	51.132(1)	52.670(1)	21.613(1)	7.928(1)
p-value			.000^*^	.000^*^	.000^*^	.000^*^	.005^*^
Residence	Urban	234(74)	38	35	32	71	74
	Rural	39(12)	64	54	54	87	85
	Peri-Urban	45(14)	29	27	22	73	80
χ^2^ (df)			12.138(2)	7.297(2)	10.058(2)	4.302(2)	2.756(2)
p-value			.002^*^	.026^*^	.007^*^	.116	.252
Systolic BP (n = 284)	Normal SBP	47(17)	28	23	19	60	66
	Prehypertension	189(67)	36	35	32	74	74
	Stage 1 HTN	29(10)	59	59	62	76	83
	Stage 2 HTN	19(7)	63	37	32	79	74
χ^2^ (df)			12.643(3)	9.717(3)	15.200(3)	4.472(3)	2.623(3)
p-value			.005^*^	.021^*^	.002^*^	.215	.453

Results were expressed as %; Fisher’s exact test was conducted when cells have expected count < 5

^*^p ≤ 0.05 was used as the limit for significance

**Table 6:** Correlation between selected independent variable with EQ-5D index and VAS

Dependent variables	EQ-5D index value		EQ-5D VAS	
Independent variables	rho	^a^p value	rho	^a^p value
Age	− .212^**^	.000	.042	.459
Sex	− .271^**^	.000	− .157^**^	.005
Monthly family expenditure (BDT)	.072	.199	.220^**^	.000
Monthly family income (BDT)	.103	.067	.234^**^	.000
Duration of Diagnosis	− .122^*^	.030	− .011	.847
PHQ 9 score	− .616^**^	.000	− .380^**^	.000
BMI (Kg/ m^2^)	.001	.986	.155^*^	.012
Education	.261^**^	.000	.205^**^	.000

^a^P values were generated using Spearman’s correlation coefficient

^*^Correlation is significant at the 0.05 level (2-tailed)

^**^Correlation is significant at the 0.01 level (2-tailed)

**Fig. 3:**
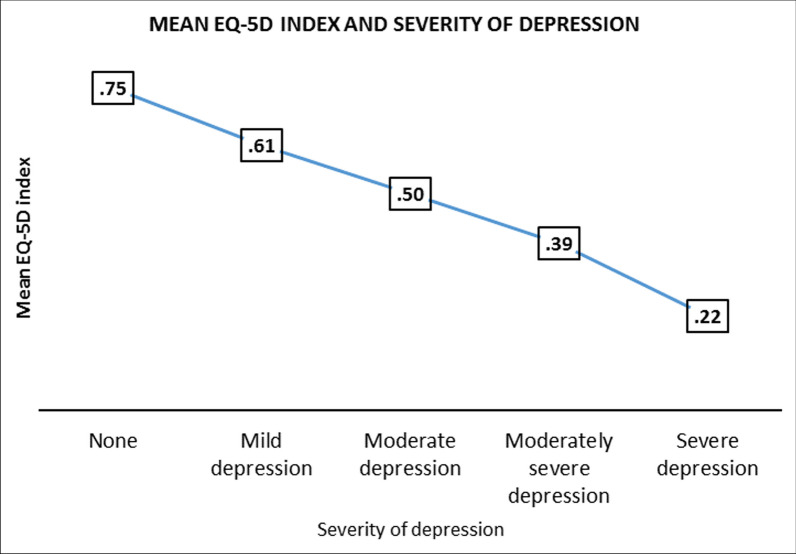
Mean EQ-5D index and severity of depression: displays the relationship between the mean EQ-5D-5L index score, a measure of HRQOL, and the severity of depression among the study population. The x-axis represents the severity of depression, measured using a validated questionnaire, and is divided into four categories: no depression, mild depression, moderate depression, and severe depression. The y-axis shows the mean EQ-5D-5L index score for each category of depression severity. The legend provides information about the mean EQ-5D-5L index score for each category of depression severity, allowing readers to interpret the relationship between depression severity and HRQOL in the study population. The graph shows a clear decrease in the mean EQ-5D-5L index score as depression severity increases, indicating that depression is associated with lower HRQOL

Table 7 presents the average PHQ-9 scores, EQ-5D index, and VAS scores based on demographic characteristics and the presence of depression. Notably, our analysis revealed that individuals with depression had significantly lower EQ-5D index values compared to those without depression (mean EQ-5D index 0.53 vs. 0.75; median 0.54 vs. 0.72; Kruskal-Wallis test *p*-value < 0.05). Moreover, the EQ-5D VAS scores were significantly lower in individuals with depression (mean EQ-5D VAS 65 vs. 76; median 70 vs. 80; Kruskal-Wallis test *p*-value < 0.05). When exploring the severity of depression and its relationship with HRQOL, the study found that the mean EQ-5D index decreased with the increasing severity of depression (Fig. 3). The relationship between the severity of depression and HRQOL was significant at the 5% confidence level (Kruskal-Wallis Test statistics = 114.84; *p* < .05).

**Table 7:** Mean PHQ 9 score, EQ-5D index and VAS by demographic characteristics (N = 318)

		N (%)	EQ-5D index Mean (SD)	EQ-5D VAS Mean (SD)	PHQ9 score Mean (SD)
Sex	Male	134(42)	.68(.19)	73(20)	5(4)
	Female	184(58)	.57(.23)	67(19)	9(5)
Age groups(years)	30–39	38(12)	.69(.21)	64(18)	7(5)
	40–49	79(25)	.64(.19)	70(21)	7(6)
	50–59	100(31)	.62(.19)	72(18)	7(5)
	60 +	101(32)	.56(.25)	68(21)	7(5)
Religion	Islam	312(98)	.61(.22)	69(20)	7(5)
	Hindu	6(2)	.64(.15)	73(17)	6(4)
Marital status	Never married	7(2)	.67(.25)	71(28)	10(8)
	Married	303(95)	.61(.22)	69(20)	7(5)
	Widow/Widower	7(2)	.69(.12)	79(11)	6(2)
	Separated/Divorced	1(1)	.72	70	9
Family History of T2DM	No	139(44)	.64(.18)	68(19)	7(5)
	Yes	179(56)	.60(.24)	70(20)	7(5)
Household members	≤ 4	145(46)	.64(.21)	70(20)	7(5)
	> 4	173(54)	.60(.22)	69(20)	8(5)
Education	No formal education	38(12)	.54(.26)	61(21)	10(6)
	Primary education	103(32)	.57(.24)	66(21)	8(5)
	Higher sec. education	108(34)	.64(.18)	72(18)	7(5)
	College and above	69(22)	.70(.18)	75(20)	5(4)
Occupation	Unemployed	3(1)	.56(.06)	65(22)	7(5)
	Service	54(17)	.68(.20)	74(23)	5(5)
	Business	32(10)	.66(.19)	72(23)	5(4)
	Labour	7(2)	.75(.22)	74(11)	8(6)
	Farming	5(2)	.63(.15)	60(10)	8(4)
	House wives	176(55)	.56(.22)	67(19)	9(5)
	Retired	41(13)	.70(.17)	74(16)	4(3)
Monthly household income(BDT)	< 10,000	52(16)	.53(.26)	63(20)	10(6)
	10,000–30000	146(46)	.63(.21)	67(20)	7(5)
	30,000–60000	83(26)	.62(.20)	73(18)	6(5)
	60,000–90000	11(3)	.69(.17)	81(14)	5(3)
	> 90,000	26(8)	.64(.19)	79(16)	6(4)
Monthly household expenditure(BDT)	< 10,000	58(18)	.56(.25)	64(19)	9(6)
	10,000–30000	155(49)	.63(.21)	68(20)	7(5)
	30,000–60000	80(25)	.62(.21)	73(19)	6(5)
	60,000–90000	9(3)	.72(.15)	83(16)	6(3)
	> 90,000	16(5)	.62(.21)	80(18)	7(4)
BMI	< 18.5 (Underweight)	9(3)	.62(.20)	66(20)	7(6)
	18.5 to < 23 (Normal wt.)	83(26)	.62(.20)	72(20)	7(5)
	23 to < 27.5 (Over wt.)	144(45)	.69(.20)	74(18)	6(6)
	≥ 27.5 (Obese)	82(26)	.55(.27)	69(27)	8(5)
Duration of diagnosis	Duration < = 3 years	108(34)	.66(.19)	70(18)	7(5)
	Duration 3–6 years	53(17)	.62(.20)	69(21)	7(5)
	Duration 6–9 years	47(14)	.59(.18)	69(18)	7(5)
	Duration > 9 years	110(35)	.58(.25)	69(22)	895)
Depressive symptoms	No depression(< 5 score)	122(38)	.75(.14)	76(16)	2(1)
	Depression(≥ 5 score)	196(62)	.53(.22)	65(21)	10(4)
Residence	Urban	234(74)	.63(.21)	71(20)	7(5)
	Rural	39(12)	.49(.27)	60(21)	10(6)
	Peri-Urban	45(14)	.67(.15)	70(18)	6(4)
Systolic BP	Normal SBP	47(17)	.72(.16)	68(23)	7(4)
	Prehypertension	189(67)	.63(.20)	71(18)	6(5)
	Stage 1 Hypertension	29(10)	.49(.25)	67(25)	10(5)
	Stage 2 Hypertension	19(7)	.57(.22)	66(19)	10(6)

## Discussion

This study examined the relationship between socio-demographic and anthropometric factors, comorbid depression, and HRQOL in adults with T2DM attending tertiary care hospitals in Dhaka, Bangladesh. The prevalence of depression and T2DM comorbid condition was 62%, which is higher than previous findings by Roy et al. [8] (34%) and Moussavi et al. [22] (9.3%). A meta-analysis by Anderson et al. [11] reported prevalence of depression ranged from 0.8 to 61% in T2DM patients. Studies in the USA and UK reported rates between 30 and 83% [54, 55], and an Iranian study found a 55% prevalence [56]. Studies from other countries indicated the prevalence of depression among T2DM patients of 37.6% in Saudi Arabia [57], 49.2% in Pakistan [25], and 17% in Dubai [58].

The higher prevalence of depression and T2DM comorbid conditions in this study may be attributed to two factors. Firstly, there was a higher proportion of women (58%) in our study compared to a previous study by Roy et al. (49%) [8]. This figure aligns with the findings from other studies that also indicated a high prevalence of depression among women [59, 60]. Second, we found a positive correlation between urban residency and depressive comorbidity. The current study had a higher proportion of urban residents (74%) than the previous study by Roy et al. (44.6%), potentially contributing to the increased prevalence of depression.

The findings of the EQ-5D in our study align with a study by Saleh et al., where most participants reported experiencing anxiety/depression, while problems in self-care and usual activities were less reported [36]. However, the proportion of patients reporting problems in EQ-5D dimensions varies across countries. A study in Indonesia found lower proportions of problems in T2DM patients without comorbid depression (mobility 37%, self-care 12%, usual activities 23%, pain/discomfort 61%, and anxiety/depression 34%) compared to those with comorbid depression (mobility 36%, self-care 20%, usual activities 36%, pain/discomfort 69%, and anxiety/depression 44%) [61]. A population-based study in China reported lower problems in EQ-5D dimensions among people diagnosed with a chronic disease in the past six months (mobility 13%, self-care 8.2%, usual activities 12.4%, pain/discomfort 28%, and anxiety/depression 15%) [62]. These variations may be attributed to differences in population health status and demographic characteristics.

Depression has a negative impact on HRQOL, particularly in patients with comorbid diabetes [63]. Studies by Wallace et al. [64], Jing et al. [65], and Cannon et al. [21] have shown that depression in T2DM patients is associated with a poorer quality of life. Our study also found a significant association between depression and all five dimensions of EQ-5D in diabetic patients. Consistent with O'Neil et al. [66], we observed that the severity of depression correlates with changes in the health state of diabetic patients. Additionally, HRQOL declines with age, potentially due to the longer duration of illness, increased mental stress, and associated complications [25, 67, 68].

The current study revealed gender differences in HRQOL, with women reporting lower HRQOL. This is consistent with the gender differences in HRQOL observed in the general population [69, 70] and among people with diabetes [57, 61]. The female gender, along with hormonal and physiological characteristics, increases the risk of developing depression and subsequently lowers HRQOL [61, 71–73]. Consistent with previous studies [64, 65, 74], HRQOL deteriorates with a longer duration of diabetes, reflecting poor glycemic control, treatment compliance [65], and the development of diabetes-associated complications over the period [75]. It is evident from previous studies that laborers score low in HRQOL compared to other occupations, mainly due to stress related to work, financial adversity, and family problems [76, 77]. In contrast, our study found higher HRQOL scores among laborers, contrasting findings from other occupations. The reasons for this discrepancy require further investigation.

The study had some limitations, including the use of purposive and convenient sampling, a shorter data collection period, and the use of instruments to assess depression and HRQOL that are not considered the gold standards. The PHQ-9 and EQ-5D rely on self-reported data and may be less accurate and sensitive for certain populations. The gold standard for assessing depression is typically a structured clinical interview using standardized diagnostic criteria, such as DSM-5 or ICD-11, conducted by trained mental health professionals. The study was cross-sectional with small samples, limiting causal or dose-response conclusions. Generalizability is restricted, as most patients were females from two hospitals in Dhaka, Bangladesh.

## Conclusions

A significant proportion of patients with T2DM in this study reported having depression and T2DM comorbid conditions. Further, depressive symptoms negatively impact the HRQOL, with the patients experiencing lower HRQOL as the severity of depression increases. These findings provide an important basis for considering early screening, diagnosis, treatment, and follow-up of depression in people with diabetes. By identifying an association between independent socio-demographic variables, depressive comorbidity, and HRQOL, this study offers scope for designing and conducting further research at a larger scale employing more rigor.

## Data Availability

All data generated or analyzed during this study are included in this published article as annexed tables.

## Abbreviations

BIHS: Bangladesh Institute of Health Science
BRAC: Bangladesh Rural Advancement Committee
BMI: Body Mass Index
CIDI: Composite International Diagnostic Interview
DMCH: Dhaka Medical College and Hospital
EuroQol-5D-5L: European Quality of Life Five Dimension and Five Level
HCDP: Health Care Development Project
HRQOL: Health-Related Quality of Life
IDF: International Diabetes Federation
NCD: Non-Communicable disease
OPD: Outpatient Department
PHQ-9: Patient Health Questionnaire (PHQ)-9
QoL: Quality of Life
SPSS: Statistical Package for Social Sciences
T2DM: Type 2 diabetes mellitus
UHC: Urban Health Centers
VAS: Visual Analogue Scale
